# Editorial: Recent Advances in Porcine Respiratory Diseases

**DOI:** 10.3389/fvets.2022.948005

**Published:** 2022-06-07

**Authors:** Małgorzata Pomorska-Mól, Arkadiusz Dors

**Affiliations:** Department of Preclinical Sciences and Infectious Diseases, Faculty of Veterinary Medicine and Animal Sciences, Poznan University of Life Sciences, Poznan, Poland

**Keywords:** PRRSV, *Actinobacillus pleuropneumoniae*, *Mycoplasma hyopneumoniae*, PRDC, respiratory diseases

This Research Topic gathers the recent data on etiological factors, pathogen-host interactions, pathogenesis, diagnosis, epidemiology and control of infectious diseases of the swine respiratory tract. Pig production is one of the fastest-growing livestock sectors in the world. Intensification of pig production and globalization are important risk factors in increasing previously uncommon diseases, changes in their spread and the emergence of new infections. Despite the enormous progress in veterinary medicine, bacterial and viral respiratory diseases in pigs still constitute a group of diseases of particular importance in the profitability of production.

This Research Topic brings together 8 articles with updated knowledge, including *Actinobacillus pleuropneumoniae* (APP), porcine reproductive and respiratory syndrome virus (PRRSV) pathology, diagnosis, epidemiology, and control and eradication and some new data regarding *Haemophilus parasuis* (currently known as *Glaesserella parasuis*).

APP is one major bacterial porcine respiratory tract pathogen causing disease outbreaks worldwide, although effective commercial vaccines are available. The first article on the Topic (Stringer, Bossé et al.) presents a quick and straightforward recombinase-polymerase-amplification (RPA) isothermal technique for the detection of APP in clinical samples. Current gold-standard diagnostic methods lack sensitivity (bacterial culture), require expensive thermocycling machinery (PCR) and are time-consuming (culture and PCR). The search for new, simple and reliable diagnostic methods is therefore welcome. The test described in this Topic achieved an excellent sensitivity (10 copies/μL), and clinical sensitivity and specificity reached 84.3 and 100%, respectively. These results indicate that the APP-RPA assay presents a suitable rapid and sensitive screening tool for this important veterinary pathogen. In the second paper on the Topic, Stringer, Li et al. described the validation of Whatman FTA^®^ cards, a specialist form of filter paper containing a chemical coating that lyses cells, denatures proteins, and binds nucleic acids, preventing their degradation by oxidation or UV radiation, for collection and processing of APP isolates, or porcine lung tissue samples, for direct use in diagnostic multiplex PCRs. This study provides simple, efficient, rapid, and practical sample processing for APP detection and molecular serotyping.

Due to the common failure of vaccination, treatment with antimicrobials is crucial to prevent animal losses within an outbreak of pleuropneumonia. To preserve the effectiveness of antimicrobial substances to fight APP should therefore be the primary aim of any interventions. Hennig-Pauka et al. present the new data on the phenotypic antimicrobial resistance in APP strains isolated in Germany from 2006 to 2020. The results presented provide optimistic data. According to the researchers, there is only a low risk of treatment failure due to resistant isolates. To prevent APP disease outbreaks in endemically infected herds more efficiently in the future, next to environmental trigger factors, preventive measures must also address the coinfecting agents.

Continuing with the problem of pleuropneumonia, Loera-Muro et al. present the evidence for the surviving of APP in biofilms in samples of drinking water from swine farms. The bacteria in the drinking water samples showed the ability to form biofilms *in vitro*. Likewise, APP biofilm formation *in situ* was observed on-farm drinkers, where the biofilm formation was in the presence of other bacteria such as *Escherichia coli, Stenotrophomonas maltophilia*, and *Acinetobacter schindleri*. These data indicate that APP can inhabit aquatic environments using multi-species biofilms as a strategy to survive outside of their host.

In the article by Nelsen et al., the results of a study on the occurrence of Porcine parvovirus 2 (PPV2) in pigs with PRDC symptoms are presented. Porcine respiratory disease complex results from multiple viral and/or bacterial co-infections. Porcine circovirus type 2 (PCV2), PRRSV, *Mycoplasma hyopneumoniae* and Influenza A viruses (IAV) are the main causative agents. PPV2 has been identified in healthy and clinically diseased pigs at a high prevalence worldwide. However, the significance of PPV2 infection in PRDC and its association with other co-infections are unclear. In the study described within the Topic, PPV2 was detected in the lung tissue in 39% of PRDC-affected pigs by qPCR. PPV2 infection was localized in alveolar macrophages and other cells in the lungs, with interstitial pneumonia in 28.2% of samples. Together, these results suggest that PPV2 is associated but may not be the sole causative agent with PRDC, warranting the control and prevention of this underdiagnosed virus.

Developing strategies to accurately predict the expected degree of disease severity before the onset of the disease is a significant challenge for farmers and veterinarians. van Dixhoorn et al. provide interesting insights into the relationships between external factors, animal-based factors, and clinical outcomes of disease. This work provides a first basis to unravel the complex network of interactions that will enable a quantitative prediction of disease outcomes in pigs and may inspire further research in this area.

In conclusion, the articles in this Research Topic discuss important features of porcine respiratory diseases and how to diagnose, prevent and control them. We hope that readers will find these articles interesting and inspiring.

## Author Contributions

MP-M and AD wrote the draft and final version of the editorial. Both authors contributed to the article and approved the submitted version.

## Conflict of Interest

The authors declare that the research was conducted in the absence of any commercial or financial relationships that could be construed as a potential conflict of interest.

## Publisher's Note

All claims expressed in this article are solely those of the authors and do not necessarily represent those of their affiliated organizations, or those of the publisher, the editors and the reviewers. Any product that may be evaluated in this article, or claim that may be made by its manufacturer, is not guaranteed or endorsed by the publisher.

